# Part-Wise Graph Fourier Learning for Skeleton-Based Continuous Sign Language Recognition

**DOI:** 10.3390/jimaging11080286

**Published:** 2025-08-21

**Authors:** Dong Wei, Hongxiang Hu, Gang-Feng Ma

**Affiliations:** College of Computer Science and Technology, Zhejiang University of Technology, Hangzhou 310023, China; hongx_hu@zjut.edu.cn (H.H.); gf_ma@zjut.edu.cn (G.-F.M.)

**Keywords:** Fourier fully connected graph, frequency enhancement, part-wise action recognition, continuous sign language recognition

## Abstract

Sign language is a visual language articulated through body movements. Existing approaches predominantly leverage RGB inputs, incurring substantial computational overhead and remaining susceptible to interference from foreground and background noise. A second fundamental challenge lies in accurately modeling the nonlinear temporal dynamics and inherent asynchrony across body parts that characterize sign language sequences. To address these challenges, we propose a novel part-wise graph Fourier learning method for skeleton-based continuous sign language recognition (PGF-SLR), which uniformly models the spatiotemporal relations of multiple body parts in a globally ordered yet locally unordered manner. Specifically, different parts within different time steps are treated as nodes, while the frequency domain attention between parts is treated as edges to construct a part-level Fourier fully connected graph. This enables the graph Fourier learning module to jointly capture spatiotemporal dependencies in the frequency domain, while our adaptive frequency enhancement method further amplifies discriminative action features in a lightweight and robust fashion. Finally, a dual-branch action learning module featuring an auxiliary action prediction branch to assist the recognition branch is designed to enhance the understanding of sign language. Our experimental results show that the proposed PGF-SLR achieved relative improvements of 3.31%/3.70% and 2.81%/7.33% compared to SOTA methods on the dev/test sets of the PHOENIX14 and PHOENIX14-T datasets. It also demonstrated highly competitive recognition performance on the CSL-Daily dataset, showcasing strong generalization while reducing computational costs in both offline and online settings.

## 1. Introduction

Sign language, characterized by its unique grammar and lexicon, enables the communication of complex ideas and emotions through coordinated body movements, including hand gestures, facial expressions, and torso movements, thus serving as an effective communication medium for the deaf community [1]. However, the majority of hearing individuals lack proficiency in sign language, necessitating the development of sign language recognition (SLR) methods to facilitate effective communication between the deaf and hearing communities [2]. SLR tasks are commonly categorized into isolated sign language recognition (ISLR) [3] and continuous sign language recognition (CSLR). The former focuses on recognizing individual sign language words—that is, Gloss—while the latter aims to identify Gloss sequences and their contextual semantics [4]. Current CSLR tasks predominantly utilize video data as input, leveraging the rich multi-modal information, including RGB images [5], optical flow [6], and human skeletons [7]. Prior to the advent of deep learning, CSLR primarily relied on handcrafted features derived from RGB data, such as HOG or SIFT [8]. Then, researchers incorporated neural networks into their designs. For instance, Cheng et al. [9] replaced handcrafted features with fully convolutional networks, and they learned spatial and temporal features from only sentence-level annotations. Han et al. [10] utilized attention mechanisms to model spatial appearance and temporal evolution concurrently. With the widespread adoption of the CTC [11] loss, which introduces blank tokens to represent uncertainties in the output sequence and allows the merging of consecutive redundant frames into a single frame, end-to-end training of models by maximizing the probability of the output sequence became feasible [12]. Although image-based methods offer abundant visual information and the potential for multi-modal fusion, the foreground and background information in SLR tasks proves to be interference. Moreover, these methods necessitate computations for all pixels or patches, leading to significant computational load.

Compared to other modalities, skeleton data contain only 2D [13] or 3D [14] human joint coordinates, making them more compact and abstract compared to other modalities. Focusing on movements, skeleton-based action-recognition methods can effectively handle complex backgrounds and varying lighting conditions, demonstrating greater efficiency and robustness. GNN-based methods have dominated skeleton-based action recognition since the introduction of ST-GCN [13]. Subsequent works, such as BlockGCN [15], have enhanced spatial modeling via learnable graph topology refinement. APLE-GCN [16] obtains part-level features by adaptively fusing the joint-level features extracted from multiple data streams. For sign language recognition tasks, Tunga et al. [17] developed a GCN-Transformer hybrid, combining GCNs [18] for spatial dependencies and Transformers [19] for temporal dynamics. The Co-sign1s [7] model partitions body parts into specialized GCN streams with inter-sequence interaction, while Co-sign2s [7] fuses skeletal and motion features. For MSKA [20], different stream-level representations within the same time period share the same semantics, and it employs multi-stream keypoint attention for feature fusion. These methods either utilize skeleton data as auxiliary information or model skeleton sequences independently, focusing solely on shallow data relationships, which fails to adequately consider multi-part coordination in actions and hinders recognition performance.

Continuous SLR presents unique challenges, due to the inherently dynamic and nonlinear nature of sign language, compounded by asynchronous body movements that complicate spatiotemporal representation. Models must account for inter-signer variability in execution speed and expressive style while accurately parsing continuous action sequences [21]. Notably, even intra-signer consistency proves challenging, as the same action may exhibit movement variations across repetitions. SignGraph [22], based on RGB data, represents sign language sequences as graphs and proposes Local Sign Graph and Temporal Sign Graph modules to learn correlations of cross-region features within frames and interactions of cross-region features between adjacent frames, respectively. Corrnet [23] proposes a novel correlation network architecture that dynamically computes spatiotemporal affinity matrices between the current frame and its adjacent temporal neighbors. In skeletal-based methods, Cosign [7] first models group-level sequences separately and then uses a complementary mask for inter-sequence interactions to obtain the final action representation. C2SLR [24] further innovates by employing skeletal heatmaps as spatial attention guides to focus on information-rich regions. The asynchrony among different parts of body actions leads to poor compatibility of spatiotemporal networks in these methods, which contradicts the unity of space and time in the real world, restricting the action representation capacity of skeletal-based methods.

**Figure 1:**
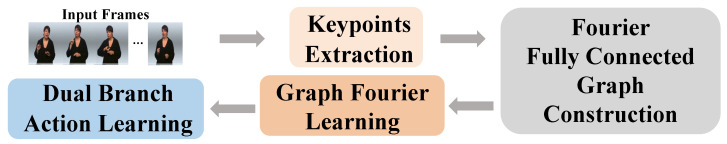
**Schematic of PGF-SLR.** Input frames are first processed by an off-the-shelf skeleton extractor to obtain skeletal data. Next, these keypoints are used to construct a Fourier fully connected graph and fed into the graph Fourier learning module for spatiotemporal representation. Finally, a dual-branch action learning module is used to learn the representations of sign language actions.

To address the aforementioned challenges, we propose a part-wise graph Fourier learning method for skeleton-based continuous sign language recognition (PGF-SLR), as shown in Figure 1. Specifically, PGF-SLR unifies spatiotemporal feature modeling from a graph learning perspective, representing both intra- and inter-part correlations of part-level features as node–node dependencies in a Fourier fully connected graph. Then, the PGF-SLR performs feature learning in the Fourier space via a graph Fourier learning module consisting of stacked Fourier graph operators (FGOs) and an adaptive feature enhancement module. Furthermore, we designed a dual-branch action learning structure comprising an action prediction assisting the sign recognition module to enhance the contextual understanding of the model. Our extensive experiments on three widely used datasets demonstrate that the PGF-SLR achieves highly competitive recognition performance using only skeletal data. The main contributions are as follows:

- We propose a novel Fourier fully connected graph action representation structure, which uses the Fourier space features of part-level topological graphs as nodes, employs inter-node attention as edges, and constructs action sequences in a globally ordered yet locally unordered manner.
- We propose a graph Fourier learning method that employs Fourier graph operators to learn representations from the Fourier fully connected graph, then applies the proposed MLP-based amplitude enhancement module to improve the sign language representation capability of the model.
- We design a dual-branch action learning strategy that integrates an action prediction branch to assist the traditional recognition branch. The two branches collaboratively reinforce each other, thereby improving the model’s understanding of sign sequences.

The rest of this paper is organized as follows: Section 2 introduces related works, Section 3 introduces the proposed PGF-SLR method, Section 4 introduces our experimental results and analysis, and Section 5 is the conclusion.

## 2. Related Work

This section provides a concise overview of related works on skeleton-based, part-based, and GNN-based action recognition.

### 2.1. Skeleton-Based Action Recognition

Advancements in pose estimation algorithms [25] and depth cameras, such as Kinect [26], have enabled the rapid and accurate acquisition of human keypoints at a low cost. Skeleton-based action recognition has garnered increasing attention, due to its more compact representation compared to RGB-based methods. Additionally, skeleton-based representations exhibit greater robustness against illumination variations and background noise. Traditional skeleton-based approaches rely on handcrafted descriptors with fixed receptive fields to model joint-level motion patterns across entire temporal sequences, which are then fed into deep networks such as RNNs or CNNs for feature extraction [27]. While these descriptors are interpretable, they typically capture only shallow and simple features, making it difficult to discover significant deep features. Inspired by graph learning, ST-GCN [13] introduced GCN to skeleton-based action recognition, leveraging the topological structure of the human skeleton to simultaneously model spatial configurations and the temporal dynamics of skeletons. CTR-GC [28] further enhanced spatial modeling through learnable topological refinement. Unlike methods that transform skeleton data into images or graphs, Transformer-based methods directly use attention blocks to model dependencies among joints. SkateFormer [29] dynamically partitions skeletal joints and temporal frames, based on four fundamental skeletal–temporal relations, performing specialized skeletal–temporal self-attention computations within each part. Shi et al. [30] adopted a pure Transformer network to explore joint relationships.

In this work, we employ skeleton sequences extracted from RGB videos using the established MMPose [25]. By computing part-level frequency attention in the Fourier space, our method overcomes data interference from RGB-based data, achieving more robust recognition results.

### 2.2. Part-Based Action Recognition

The human body is a natural topological structure [31], and part-based action recognition methods aim to learn action representations from the coordination of different body parts. Du et al. [32] proposed a hierarchical recurrent neural network for hierarchically concatenating features from different body parts. Chen et al. [33] proposed a coarse-to-fine framework that first predicts the video-level action category and then localizes body parts for part-level action recognition. Song et al. [34] proposed a part attention mechanism to identify the most informative parts. The MGCF-Net [35] model first segments the input into clips and then divides these clips into several parts, based on the structure of the human body. It employs multi-head self-attention to capture contextual features at both the joint and part levels. MMP-ST [36] is a multimodal method incorporating both skeletal part features and textual features for action recognition. STF-Net [37] proposed a multi-grain contextual focus module that captures multi-scale features and a temporal discrimination focus module to capture the local sensitive points of the temporal dynamics. GAP [38] proposed a multimodal training scheme that employs a pre-trained large-scale language model as the knowledge base to generate prompts for body parts and supervise the action encoder. All these methods adopt complex strategies for individual information propagation or fusion of information from different parts.

Considering the nonlinearity of sign language and the asynchrony between parts, we partition sign language actions into parts and use part-level Fourier features as nodes in a Fourier fully connected graph to understand signs in a globally ordered yet locally unordered manner.

### 2.3. GNN-Based Action Recognition

The existing GNN-based action recognition methods heavily rely on predefined graph structures to establish spatial correlations. However, these methods fail to capture spatial correlation patterns over time. Recognizing the limitations of fixed topology based on natural connections, subsequent works have adopted learnable topologies for action recognition. Notably, CTR-GC [28] is a spatial-adaptive graph generation method based on non-local mechanisms to enhance the flexibility of the skeletal graph structure, thereby improving GNN performance. The GTC-Net [39] model is a complementary network integrating GCN and Transformer to capture both local and global joint correlations, enabling parallel information interaction between the two domains. STA-GCN [40] is a spatial-temporal adaptive GCN that learns an adaptive integrate strategy for spatial and temporal features. DS-GCN [41] is a joint-type-aware and edge-type-aware adaptive topology that integrates these semantic modules with temporal convolution for skeleton-based action recognition. JDP-GCN [42] proposed a distributed spatiotemporal perception module for joint-wise distributed multi-location perception and an anchor pose-driven subaction encoding module for informative clues for subaction reasoning. However, these decoupled modeling approaches, which independently learn spatial and temporal correlations, contradict unified spatiotemporal dependencies in real-world scenarios, ultimately limiting their predictive performance.

Recent research has explored combining complex numbers with graph structures, particularly through Complex-Valued Neural Networks [43] (CVNNs) that process complex inputs and parameters to transform signals between time–frequency domains, efficiently handling phase and amplitude components via complex matrix operations [44]. Graph extensions of time–frequency analysis [45] examine localized spectral features around vertices using window functions, while architectures like GrokFormer [46] integrate graph Fourier transforms with Kolmogorov–Arnold networks to learn adaptive spectral representations. However, the direct modification of complex numbers in traditional frequency domain methods may cause phase distortion.

In this paper, we propose a novel graph Fourier learning method that constructs multi-part action sequences from a pure graph perspective. Our approach uniformly models spatiotemporal dependencies through Fourier fully connected graphs and efficiently performs matrix multiplication in the frequency domain using FGOs.

## 3. Methodology

We propose a part-wise graph Fourier learning method for skeleton-based continuous sign language recognition. The method constructs a unified dependency representation by modeling part-level features as nodes and their frequency domain attention as edges, effectively capturing skeletal motion patterns to boost recognition accuracy.

### 3.1. Problem Definition

**Figure 2:**
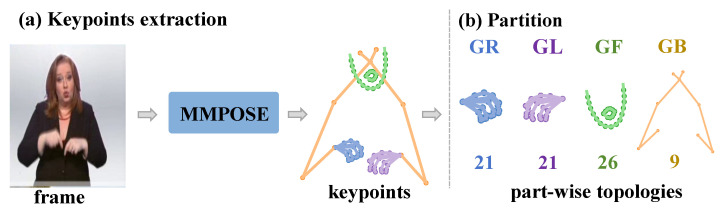
**Skeleton extraction and partition:** (**a**) skeleton extraction from sign language frames using MMPose; (**b**) keypoints are anatomically partitioned into four topological graphs: GR (right-hand with 21 nodes), GL (left-hand with 21 nodes), GF (face with 26 nodes), and GB (torso with 9 nodes).

As shown in Figure 2, we employ the well-established skeleton-extraction method to extract human skeletal data from sign language videos, and we focus on 77 nodes of the upper body and segmenting them into four parts: the torso, with 9 nodes; both hands, with 21 nodes; and the face, with 26 nodes; these four parts are denoted as GB, GL, GR, and GF for the corresponding part-level topological graphs. Let $X\in R^{T\times 77\times 3}$ represent a skeletal sequence with *T* frames and 3 channels. Our objective is to accurately model part-level feature interactions in Fourier space to obtain higher-quality action representations, thereby enhancing the model’s capability for sign language recognition.

### 3.2. Model Framework

**Figure 3:**
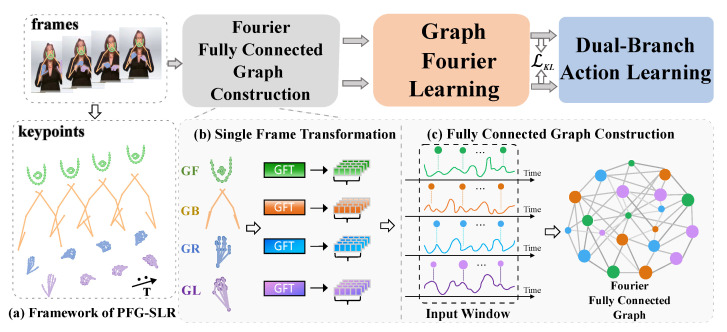
**Framework of PGF-SLR.** The PGF-SLR consists of a Fourier fully connected graph construction module, a graph Fourier learning module, and a dual-branch action learning module, which includes a recognition branch and an auxiliary prediction branch (**a**). The Fourier fully connected graph is constructed as (**b**) skeletons are segmented to four parts and transformed to Fourier Space by GFT, and (**c**) part-level features of frames within an input window are treated as nodes and their attention are treated as edges to construct the Fourier fully connected graph.

As shown in Figure 3, the PGF-SLR consists of three main modules:

- Fourier fully connected graph construction module. This module first maps part-level topology graphs into Fourier space using graph Fourier transformation (GFT), and it constructs a Fourier fully connected graph using the part-level Fourier representations as nodes features and their frequency domain attention as edges.
- Graph Fourier learning module. The module employs stacked Fourier graph operators (FGOs) to learn the spatiotemporal relationships among nodes in the Fourier fully connected graph. An adaptive frequency enhancement module is attached to enhance the learned action features.
- Dual-branch learning module. This module comprises an auxiliary action prediction branch to assist the sign language recognition branch to obtain higher-quality sign language action representations.

### 3.3. Fourier Fully Connected Graph Construction

Due to the nonlinear characteristics of sign language, asynchronous movements naturally occur during multi-part coordination. Unlike the classical two-stage approach that independently models spatial and temporal information [20], the Fourier fully connected graph learns action representations from the perspective of graph learning. It unifies the intra- and inter-sequence correlations of part-level features as node dependencies in a fully connected graph, eliminating the incompatibility of spatiotemporal modeling. This approach constructs adaptive spatiotemporal dependencies, facilitating the learning of asynchrony among actions.

The subgraphs in Figure 3b,c illustrate the Fourier fully connected graph construction process. Specifically, each topology graph is defined as $G_{p}=\left(X_{p},A_{p}\right)$, with $A_{p}$ representing the part-level anatomical adjacency matrix and nodes $X_{p}\in \left(x,y,c\right)$ representing skeletal joints, including (x,y) coordinates and confidence value *c*. We first utilize the graph Fourier transform (GFT) to transform the $G_{p}$ of the four parts into Fourier space, obtaining four part-level Fourier features denoted as $f_{GB}\in R^{d}$, $f_{GL}\in R^{d}$, $f_{GR}\in R^{d}$, and $f_{GF}\in R^{d}$, where *d* is the dimension of the Fourier features, as shown in Figure 3b. Given an input window $W_{in}$ at time step *t*, the input feature is denoted as $X_{t}^{W_{in}}\in R^{P\times W_{in}\times d}$, where *P* is the number of parts. We use the part-level Fourier features as nodes and the frequency domain attention among the nodes as edges, i.e., the values at the corresponding positions in the adjacency matrix $A_{t}^{W_{in}}\in R^{\left(P\times W_{in}\right)\times \left(P\times W_{in}\right)}$, to construct the corresponding Fourier fully connected graph $G_{t}^{W_{in}}=\left(X_{t}^{W_{in}},A_{t}^{W_{in}}\right)$, as shown in Figure 3c. Thus, we can formulate the part-wise action understanding task as the understanding of the fully connected graph and formalize it as

(1) $${\hat{Y}}_{t}=F_{\theta_{G}}\left(X_{t}^{G},A_{t}^{G}\right)$$

where $F_{\theta_{G}}$ is the graph Fourier learning module and $\theta_{G}$ represents the parameter of the module.

### 3.4. Graph Fourier Learning

The graph Fourier learning module consists of a Fourier graph neural network and an adaptive frequency enhancement module.

#### 3.4.1. Fourier Graph Neural Network

**Figure 4:**
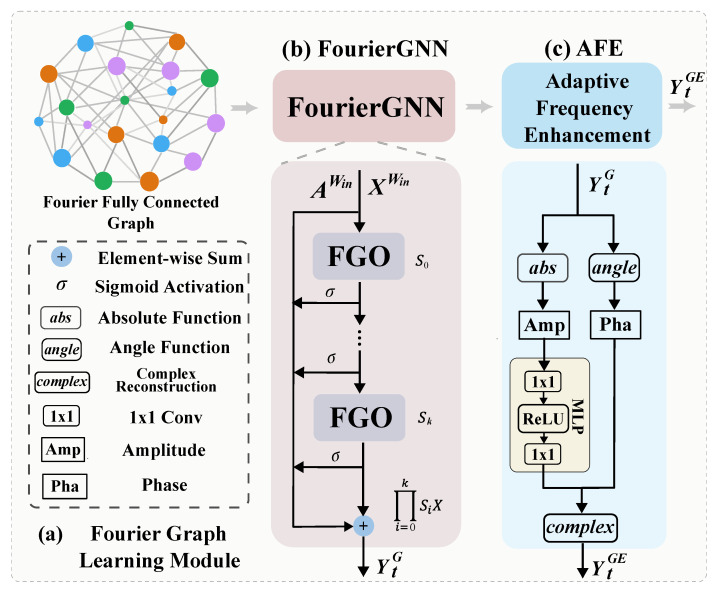
**Graph Fourier learning module.** This module consists of a FourierGNN module and an adaptive frequency enhancement module (AFE), as shown in (**a**), while in (**b**) the FourierGNN is composed of multiple stacked Fourier graph operators (FGOs), and the output $Y_{t}^{G}$ is obtained by adding together the iteratively multiplied mediate outputs; in (**c**), the adaptive frequency enhancement module contains a phase branch and an amplitude branch. The amplitude branch is adaptively enhanced via an 1×1 convolution-based MLP and then recombined with the phase to reconstruct the complex number, yielding the frequency-enhanced feature $Y_{t}^{GE}$.

Representing action sequences as pure graphs can enhance spatiotemporal modeling. Given a Fourier fully connected graph $G^{W_{in}}=\left(X^{W_{in}},A^{W_{in}}\right)$, the size of the graph grows with the window size $W_{in}$, resulting in a quadratic increase in computational cost for classical graph networks, and it poses optimization challenges when obtaining precise hidden node representations [31]. To address these issues, by defining a weight matrix $W\in R^{d\times d}$ we introduce a learnable Fourier graph operator (FGO) [47] based on a tailored Green’s kernel $\kappa :\left[P\times W_{in}\right]\times \left[P\times W_{in}\right]\to R^{d\times d}$, where $\kappa \left[m,n\right]=A_{mn}^{W_{in}}⊙W$ and κ[m,n]=κ[m−n], which ensures translation invariance, thereby enabling the model to capture part-level relationships in the Fourier domain of fully connected graphs, as is shown in Figure 4b. We then define the FGO as $S_{G}=F\left(\kappa \right)\in C^{P\times W_{in}\times d\times d}$, where F() denotes the Discrete Fourier Transform (DFT).

According to the convolution theorem [48], the Fourier transform of the convolution of two signals is equivalent to the product of their Fourier features in the frequency domain. Thus, in the Fourier space the multiplication of $F(X^{W_{in}})$ with the FGO can be expressed as

(2) $$\begin{array}{cc} F\left(X^{W_{in}}\right)F\left(\kappa \right) & =F\left(\left(X^{W_{in}}*\kappa \right)\left[m\right]\right)=F\left(\sum_{n=1}^{P\times W_{in}}X^{W_{in}}\left[n\right]\kappa \left[m-n\right]\right)\\ \; & =F\left(\sum_{n=1}^{P\times W_{in}}X^{W_{in}}\left[n\right]\kappa \left[m,n\right]\right),\forall m\in \left[0,1,..,W_{in}\right] \end{array}$$

where $\left(X^{W_{in}}*\kappa \right)\left[m\right]$ represents the convolution of *X* and κ. Based on the definition $\kappa \left[m,n\right]=A_{mn}^{W_{in}}⊙W$, the convolution of Equation (2) can be expressed as

(3) $$\sum_{j=1}^{P\times W_{in}}X^{W_{in}}\left[n\right]\kappa \left[m,n\right]=\sum_{j=1}^{P\times W_{in}}A_{mn}X^{W_{in}}\left[n\right]W=AXW$$

Thus, we can derive

(4) $$F\left(X^{W_{in}}\right)S_{G}=F\left(AX^{W_{in}}W\right)$$

indicating that performing the multiplication of $F(X^{W_{in}})$ and the Fourier-transformed Green’s kernel in the Fourier space corresponds to a graph convolution operation in the time domain. However, multiplication in the Fourier space has significantly lower complexity compared to shifting operations in the time domain $\left(O\left(n^{2}\right)>O\left(n\right)\right)$. Thus, we employ an *n*-invariant polynomial Fourier kernel $S\in C^{d\times d}$ to reduce computational complexity. During the feature learning stage, by stacking multiple layers of FGOs we recursively multiply $X_{t}^{W_{in}}$ and the FGO operator $S_{0:k}$ in the Fourier space and represent the output as

(5) $$Y_{t}^{G}=\sum_{k=0}^{K}\sigma (F\left(X^{W_{in}}\right)S_{0:k}+b_{k}),\;\;\;S_{0:k}=\prod_{i=0}^{k}S_{i}$$

where σ is a nonlinear activation function, which introduces the non-linear graph information diffusion ability to model during the summation process, $S_{k}$ is the FGO operator at the *k*-th layer, and $b_{k}\in C^{d}$ is the bias. Since all the operations are performed in the Fourier space, all the parameters are complex numbers.

#### 3.4.2. Adaptive Frequency Enhancing Module

To effectively capture the detailed characteristics of motion sequences while avoiding the phase distortion caused by direct complex number modification in traditional frequency domain methods [43], we enhance the frequency domain features processed by FourierGNN through selective frequency band amplification with strict phase relationship preservation, as shown in Figure 4c.

To be specific, we first decompose the complex spectrum $Y_{t}^{G}$, as follows:

(6) $$Amp_{Y}=\left|Y_{t}^{G}\right|$$

(7) $$Pha_{Y}=angle\left(Y_{t}^{G}\right)$$

where the amplitude spectrum $Amp_{Y}$ is adaptively enhanced to amplify critical frequency components, while the phase spectrum $Pha_{Y}$ is preserved to maintain structural information. For the amplitude spectrum, we perform nonlinear transformation, using a 1×1 convolution-based MLP to adaptively enhance the expressive ability of the key frequency components:

(8) $$Amp_{Y}^{\prime }=\sigma \left(W_{2}\cdot ReLU\left(W_{1}\cdot Amp_{Y}+b_{1}\right)+b_{2}\right)$$

where $W_{1}\in R^{\left(P\times W_{in}\right)\times \left(2\times P\times W_{in}\right)}$ and $W_{2}\in R^{\left(2\times P\times W_{in}\right)\times \left(P\times W_{in}\right)}$ are the learnable weight matrices, and where $b_{1}\in R^{2\times P\times W_{in}}$ and $b_{2}\in R^{P\times W_{in}}$ are the corresponding biases of the two convolution layers. The enhanced amplitude spectrum $Amp_{Y}^{\prime }$ is then recombined with the original phase spectrum $Pha_{Y}$ to reconstruct the enhanced complex frequency $Y_{t}^{GE}$ representation, as follows:

(9) $$Y_{real}=Amp_{Y}^{\prime }\cdot \cos\left(Pha_{Y}\right)$$

(10) $$Y_{imag}=Amp_{Y}^{\prime }\cdot \sin\left(Pha_{Y}\right)$$

where $Y_{real}$ and $Y_{imag}$ represent the real part and the imaginary coefficient of the adaptively enhanced frequency representation, and where cos() and sin() denote the cosine and sine function. Overall, the pipeline of the graph Fourier learning module is shown in Algorithm 1.
**Algorithm 1:** Graph Fourier learning.**  Input**: Fourier fully connected graph $G^{W_{in}}=\left(X^{W_{in}},A^{W_{in}}\right)$**  Output**: Enhanced action representation $Y_{t}^{GE}$**1 for** i=0 **to** *k* **do****2** $\lfloor Y_{t}^{G}=Y_{t}^{G}+\sigma \left(F\left(X^{W_{in}}\right)S_{i}+b_{i}\right)$;
// *k* stacked FGOs
**3** $Amp_{Y}=abs\left(Y_{t}^{G}\right)$;
// Get amplitude**4** $Pha_{Y}=angle\left(Y_{t}^{G}\right)$;
// Get phase
**5** $Amp_{Y}^{\prime }=\sigma \left(W_{2}\cdot ReLU\left(W_{1}\cdot Amp_{Y}+b_{1}\right)+b_{2}\right)$;

// 1×1 conv based MLP
**6** $Y_{real}=Amp_{Y}^{\prime }\cdot cos\left(Pha_{Y}\right)$;
// Enhanced real part
**7** $Y_{imag}=Amp_{Y}^{\prime }\cdot sin\left(Pha_{Y}\right)$;

// Enhanced image coefficient
**8** $Y_{t}^{GE}=complex\left(Y_{real},Y_{imag}\right)$;

// Frequency-enhanced representation $Y_{t}^{G}E$**9 return**$\;Y_{t}^{G}E$



### 3.5. Dual-Branch Action Learning Module

While humans can recognize sign language from just a few key motions, current sign language recognition methods still require processing massive amounts of video frames, resulting in significant redundancy. Previous studies [49] demonstrate that keyframes account for merely 45%, 47%, and 42% of the PHOENIX14, PHOENIX14-T, and CSL-Daily datasets, respectively, with adjacent frames showing exceptionally high similarity. Thus, in this study, we segment the input data into two keyframe subsequences. Specifically, adjacent frames in the input sequence $X\in R^{T\times 77\times 3}$ are grouped into pairs, with each frame randomly assigned to one of the two subsequences, resulting in $X_{1}\in R^{T/2\times 77\times 3}$ and $X_{2}\in R^{T/2\times 77\times 3}$. The outputs of these two keyframe subsequences, after undergoing Fourier fully connected graph learning, are denoted as $Y_{t1}^{G}$ and $Y_{t2}^{G}$, serving as inputs to the subsequent dual-branch action learning module. Specifically, $Y_{t1}^{G}$ is used for sign language context learning, while $Y_{t2}^{G}$ is utilized for action prediction. We then align them, using Kullback–Leibler (KL) divergence, yielding the following loss:

(11) $$L_{KL}=\frac{1}{2}\left(KL\left(Y_{t1}^{G},Y_{t2}^{G}\right)+KL\left(Y_{t2}^{G},Y_{t1}^{G}\right)\right)$$

#### 3.5.1. Sign Language Recognition Branch

**Figure 5:**
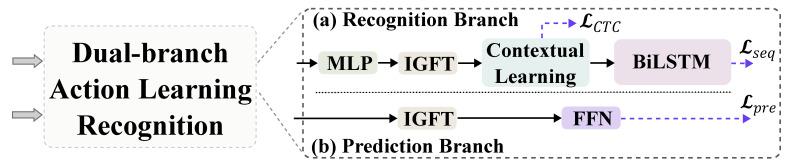
**Dual-branch action learning module.** The module consists of two parallel branches: (**a**) the recognition branch in the upper part, and (**b**) the prediction branch in the lower part.

We first transform the input to the sign language recognition branch, $Y_{t1}^{G}$, to the temporal domain by using inverse graph Fourier transform (IGFT), obtaining $Y_{t1}^{IG}\in R^{P\times W_{in}\times d}$. We then use a two-layer MLP to transform and flatten it to ${\hat{Y}}_{t1}\in R^{1\times (W_{in}\times d)}$. Referring to models [4,50] taking VAC [2] as a backbone, we also attach a 1D-CNN-based sign language contextual learning module and a BiLSTM-based [51] long-term sequence learning module, and we use two CTC-based losses, $L_{CTC}$ and $L_{seq}$, to supervise the short- and long-term feature learning of the sign language recognition branch, as is shown in Figure 5a.

#### 3.5.2. Action Prediction Branch

The action prediction branch forecasts the values for the next τ time steps ${\hat{Y}}_{t2}=\left[x_{t+1},\;\ldots ,x_{t+\tau }\right]$, based on the feature values $X_{t2}=\left[x_{t-W_{in}+1},\;\ldots ,x_{t}\right]$ within the current input window of $W_{in}$ time steps, where $x_{t}\in R^{P\times d}$. As shown in Figure 5b, after obtaining the final graph representation $Y_{t2}^{G}$, we use the IGFT to transform it back to the time domain, which yields $Y_{t2}^{IG}$. We then project $Y_{t2}^{IG}$ through a Feed Forward Network (FFN) with two layers linear of transformation with ReLU activations to obtain the predictions for the next τ time steps, which can be formalized as

(12) $${\hat{Y}}_{t2}=ReLU\left(Y_{t2}^{IG}W_{3}+b_{3}\right)W_{4}+b_{3}$$

where $W_{3}\in R^{\left(P\times W_{in}\times d\right)\times d_{1}^{FFN}}$ and $W_{4}\in R^{d_{1}^{FFN}\times d^{\tau }}$ are the weights and $b_{3}\in R^{d_{1}^{FFN}}$, $b_{4}\in R^{d^{\tau }}$ are the biases of the two layers, respectively, and where $d_{1}^{FFN}$ and $d^{\tau }$ represent the dimensions of the two layers. Using $Y_{t2}$ as ground truth, the loss of the prediction branch is

(13) $$L_{pre}=\sum_{i}^{\tau }\left|\right|Y_{t2}^{i}-{\hat{Y}}_{t2}^{i}{\left|\right|}_{2}^{2}$$

### 3.6. Loss Function

To achieve better training results, we propose a new loss function:

(14) $$L=L_{seq}+L_{CTC}+\alpha_{1}L_{KL}+{\alpha_{2}L}_{pre}\;$$

where $L_{seq}$ and $L_{CTC}$ are the traditional losses based on the VAC [2] model, $L_{KL}$ is the alignment loss for two keyframe subsequences, $L_{pre}$ is the action prediction loss, and $\alpha_{1}$ and $\alpha_{2}$ are the weights for $L_{KL}$ and $L_{pre}$, respectively.

## 4. Experiments

This section details our experiments, including the datasets, implementation details, baseline methods and evaluation metric. We benchmark our method against state-of-the-art approaches and provide comprehensive empirical analysis.

### 4.1. Datasets

We selected three of the most commonly used and representative datasets in the field of sign language recognition for our comparative experiments:

- PHOENIX14 [52] is a dataset recorded by nine presenters, extracted from German weather forecasts with high-contrast backgrounds. It contains 6841 sentences with a total of 1295 Glosses. The dataset is divided into train/dev/test sets, comprising 5672/540/629 samples, respectively.
- PHOENIX14-T [1] is another dataset extracted from German weather forecasts. It includes 1085 Glosses distributed across 8247 sentences. The distribution of samples in train/dev/test sets is 7096/519/642, respectively.
- CSL-Daily [53] is a Chinese Sign Language dataset related to daily life, recorded indoors by 10 signers. Compared to the previous two datasets, it has a noisier background. The dataset consists of 20654 sentences, with the train/dev/test sets containing 18401/1077/1176 samples, respectively.

### 4.2. Implementation Details

**Figure 6:**
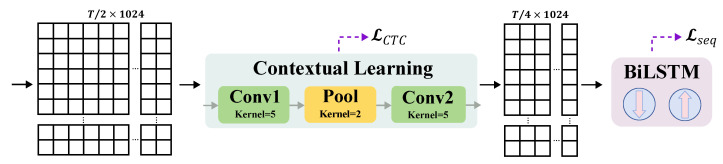
**Implementation of contextual learning module and long-term sequence modeling module.** The contextual learning module consists of a 1-D convolution with kernel size 5, a 1-D max-pooling with kernel size 2, and a second 1-D convolution with kernel size 5, supervised by the $L_{CTC}$. The long-term sequence modeling module comprises a two-layer BiLSTM with a hidden size of 1024, supervised by $L_{seq}$.

For this paper, we used the well-established MMPOSE [25] to extract human skeletal information from RGB-based datasets. Following VAC [2], the short-term temporal convolution of the sign language recognition branch of PGF-SLR consists of a convolution with a kernel size of 5, a max-pooling with a kernel size of 2, and a second convolution with a kernel size of 5, as shown in Figure 6. For long-term sequence modeling, a two-layer Bidirectional Long Short-Term Memory (BiLSTM) network with a hidden layer size of 1024 was employed. The weights for the loss terms $L_{KL}$ and $L_{pre}$ were set to $\alpha_{1}=1.0$ and $\alpha_{2}=0.2$, respectively. All our experiments were conducted on an Intel(R) Xeon(R) Platinum 8352V CPU platform with a single NVIDIA-A6000 48G GPU, using a batch size of 1.

### 4.3. Baseline Methods

To evaluate the proposed method, we selected the state-of-the-art and most representative method from three types of sign language recognition models using different input modalities as the baseline methods: (1) models using only RGB input data, including VAC [2], SMKD [54], TLP [55], AdaBrowse [56], Contrastive [57], SEN [58], HST-GNN [59], TCNet [60], and SignGraph [22]; (2) models using keypoints and other modalities, including STMC [61], Cosign-2s [7], and C2SLR [24]; (3) models using only keypoints data, including Cosign-1s [7] and MSKA [20].

### 4.4. Evaluation Metrics

For this paper, we used the Word Error Rate (WER) [62] as the evaluation metric, defined as the minimum sum of substitution (#sub), insertion (#ins), and deletion (#del) operations required to transform the predicted sentence into the ground truth (#reference). A lower WER indicates higher accuracy:

(15) $$\mathrm{WER}=\frac{\#sub+\#ins+\#del}{\#reference}$$

### 4.5. Comparison with Baseline Methods

Table 1 and Table 2 present our experimental comparison of the PGF-SLR with the baseline models. The experimental results demonstrate that the PGF-SLR performed well across all three datasets. On the PHOENIX14 dataset, the PGF-SLR surpassed all the baseline models, yielding relative gains of 3.31% on dev and 3.70% on test over the previous SOTA TCNet. Similarly, on the PHOENIX14-T dataset the PGF-SLR achieved the highest recognition accuracy, outperforming SignGraph by relative improvements of 2.81% and 7.33% on the dev and test sets, respectively. As can be seen in Table 2, though slightly below that of the SignGraph, our model still achieved competitive recognition accuracy on the more complex CSL-Daily dataset. Compared to the models that used only skeleton input, the PGF-SLR showed significant improvements in recognition capability and outperformed most of the models that used RGB or multimodal inputs. Overall, the PGF-SLR demonstrated considerable recognition performance across all three datasets, highlighting the strong generalization ability of the part-wise graph Fourier learning method for skeleton-based continuous sign language recognition.

### 4.6. Ablation Study

To systematically evaluate the contributions of the different components in our PGF-SLR model, we conducted comprehensive ablation studies, using CoSign-1s as the baseline model. The experiments examined three key components: DI (dual-keyframe subsequences input), FGL (Fourier fully connected graph construction and graph Fourier learning), and PRE (action prediction branch). As can be seen in Table 3, in control group a_1, training on keyframe subsequences alone yielded a modest improvement over the baseline. In a_2, applying the FGL module to the full sequence produced substantial gains, confirming its strong motion-learning capacity. In experimental group a_3, adding PRE in isolation yielded only limited improvement. In a_4 and a_6, we combined FGL with DI and PRE, respectively; both configurations surpassed a_2. However, the DI–PRE pair without FGL in a_5 delivered only marginal gains, indicating that DI and PRE are most effective when integrated with FGL. When all three modules were employed, the PGF-SLR achieved optimal performance, significantly outperforming the baseline across all metrics on all datasets. These results demonstrate that each of the three proposed modules contributes positively to the model’s recognition capability.

### 4.7. Hyperparameter Sensitivity Analysis

#### 4.7.1. Weights of Loss Function

**Figure 7:**
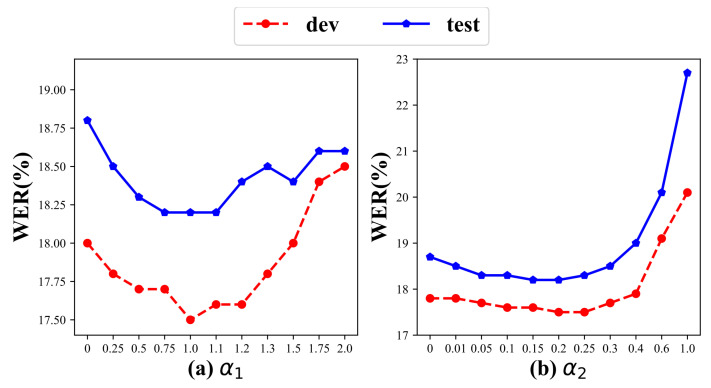
Hyperparameter sensitivity analysis of the performance trends of weights in the loss function on the PHOENIX14 Dataset: (**a**) illustrates the impact of varying the weight $\alpha_{1}$ on model recognition performance, while (**b**) depicts the impact of adjusting $\alpha_{2}$.

As shown in Figure 7, we analyzed the impact of $\alpha_{1}$ and $\alpha_{2}$ in the loss function. When analyzing $\alpha_{1}$, $\alpha_{2}$ was set to 0.2; conversely, $\alpha_{1}$ was set to $\alpha_{2}$ when analyzing $\alpha_{2}$. The trends of the same parameter were consistent across the dev and test sets. Due to gradient accumulation in the recognition module, the model was more sensitive to $\alpha_{2}$ and the value of $L_{pre}$ was relatively large, making the model more sensitive to changes in $\alpha_{2}$ and more robust against $\alpha_{1}$. As shown in Figure 7a, the model’s performance improved as $\alpha_{1}=1$. For $\alpha_{2}$, larger values significantly affected recognition capability, with the best performance at $\alpha_{2}=0.2$, as shown in Figure 7b. Therefore, we selected $\alpha_{1}=1$ and $\alpha_{2}=0.2$ as the weights for the loss function.

#### 4.7.2. Input Window Size

**Figure 8:**
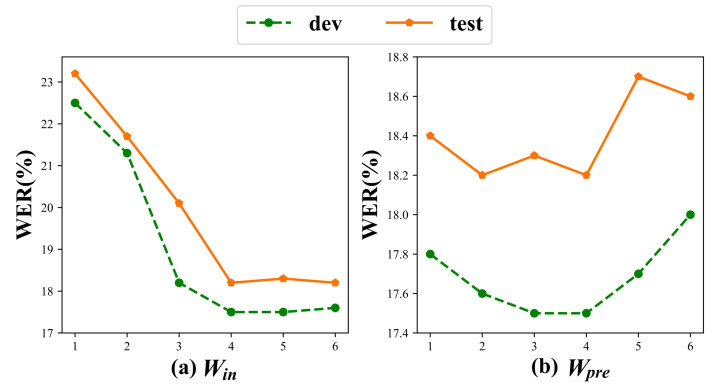
Hyperparameter sensitivity analysis of the effects of different window sizes: (**a**) demonstrates the performance changes with varying input window sizes $W_{in}$, while (**b**) illustrates the impact of different prediction window sizes $W_{pre}$.

We conducted six experimental groups with varying window sizes, $W_{in}=\left\{1,2,3,4,5,6\right\}$, to analyze their impact on action representation and recognition performance, where $W_{in}$ denotes the input window size. As shown in Figure 8a, the smaller input windows were unable to effectively capture the relationships between actions. As the window size increased, the model was exposed to more features, which led to more efficient representation and higher recognition accuracy. However, once the window size reached a certain threshold, further enlargement $\left(W_{in}>4\right)$ yielded no additional accuracy gains. Therefore, we ultimately selected an input window size of 4 for constructing the fully connected graph.

#### 4.7.3. Prediction Window Size

We conducted extensive experiments on the PHOENIX14 dataset with six different window size configurations $W_{pre}=\left\{1,2,3,4,5,6\right\}$ for the action prediction branch. Here, $W_{pre}$ denotes the prediction window size. The experimental results in Figure 8b reveal that window size significantly affected recognition accuracy, where both excessively large and small windows degraded performance. Specifically, the action prediction achieved optimal recognition performance when $W_{pre}=4$, striking an effective balance between sufficient temporal context and computational efficiency. Ultimately, we adopted $W_{pre}=4$ as the prediction steps, as it provided the best trade-off between recognition accuracy.

### 4.8. Online Inference

Unlike offline training, real-world online applications require models to process video frames sequentially and perform real-time recognition using both current and historical temporal information. We designed online recognition experiments to test the effectiveness of our PGF-SLR model in practical applications, and AdaBrowse [56] was selected as the baseline model. AdaBrowse is a lightweight RGB-based sign language recognition model that accelerates inference by pruning redundant visual data; among the state-of-the-art methods, it achieves the lowest GFLOPs and highest Throughput, rendering it exceptionally well-suited for real-time deployment. We directly used the skeleton sequences extracted from the PHOENIX14 dataset to simulate real-world scenarios. Three metrics were selected to analyze the online inference ability of the PGF-SLR: Throughput (videos/second), GFLOPs (Giga Floating-Point Operations per Second), and WER. As shown in Table 4, our PGF-SLR significantly outperformed the baseline in all metrics, delivering a 62.37%-faster inference speed and yielding a 15% relative WER reduction, demonstrating good recognition accuracy with skeleton data. These results validate the effectiveness of our PGF-SLR in practical applications.

## 5. Conclusions

We propose a part-wise Fourier fully connected graph learning method for continuous sign language recognition, exploring skeleton-only sign language recognition in the Fourier space. First, we partition the skeleton data into multiple parts and obtain their Fourier domain features via graph Fourier transform. These features serve as node representations in a fully connected graph, with their inter-part relationships modeled as edges. In the graph Fourier learning module, we sequentially apply the Fourier graph neural network and the adaptive frequency enhancement module to refine action representations by amplifying phase and amplitude information. Finally, a dual-branch action learning module, where an action prediction branch assists the sign recognition branch, enables collaborative learning, yielding deep comprehension of skeleton-based sign sequences. Our extensive experiments on the three most widely used sign language datasets confirmed that our PGF-SLR using purely skeleton features is both efficient and robust. Specifically, the PGF-SLR attained relative improvements of 3.31%/3.70% on the dev/test sets of PHOENIX14 and 2.81%/7.33% on PHOENIX14-T over the prior state-of-the-art method, while delivering highly competitive accuracy on the CSL-Daily dataset. What is more, the PGF-SLR also achieved a 62.37% relative improvement of Throughput. In the future, we will investigate multimodal continuous sign language recognition leveraging skeleton data alongside other modalities.

## Figures and Tables

**Table 1:** Comparison of PGF-SLR with baseline models on the PHOENIX14 and PHOENIX14-T datasets.

Methods	Backbone	PHOENIX14				PHOENIX14-T	
		dev (%)		test (%)		dev (%)	test (%)
		del/ins	WER	del/ins	WER		
VAC [2]	ResNet18	7.9/2.5	21.2	8.4/2.6	22.3	-	-
SMKD [54]	ResNet18	6.8/2.5	20.8	6.3/2.3	21.0	20.8	22.4
TLP [55]	ResNet18	6.3/2.8	19.7	6.1/2.9	20.8	19.4	21.2
AdaBrowse [56]	ResNet18	6.0/2.5	19.6	5.9/2.6	20.7	19.5	20.6
Contrastive [57]	ResNet18	5.8/2.6	19.6	5.1/2.7	19.8	19.3	20.7
SEN [58]	ResNet18	5.8/2.6	19.5	7.3/4.0	21.0	19.3	20.7
HST-GNN [59]	ST-GCN	-	19.5	-	19.8	19.5	19.8
SignGraph [22]	Custome(GCN)	6.0/2.2	18.2	5.7/2.2	19.1	17.8	19.1
TCNet [60]	ResNet18	5.5/2.4	18.1	5.4/2.0	18.9	18.3	19.4
STMC [61]	VGG11	7.7/3.4	21.1	7.4/2.6	20.7	19.6	21.0
C2SLR [24]	ResNet18	6.8/3.0	20.5	7.1/2.5	20.4	20.2	20.4
CoSign-2s [7]	ST-GCN	-	19.7	-	20.1	19.5	20.1
TwoStream-SLR [63]	ST-GCN	-	18.4	-	18.8	17.7	19.3
MSKA [20]	ST-GCN	-	21.7	-	22.1	20.1	20.5
CoSign-1s [7]	ST-GCN	-	20.9	-	21.2	20.4	20.6
PGF-SLR	FourierGNN	4.4/2.7	17.5	4.2/2.9	18.2	17.3	17.7

**Table 2:** Comparison of PGF-SLR with baseline models on the CSL-Daily dataset.

Methods	dev (%)	test (%)
SEN [58]	31.1	30.7
TCNet [60]	29.7	29.3
SignGraph [22]	26.4	25.8
CoSign-2s [7]	28.1	27.2
MSKA [20]	28.2	27.8
CoSign-1s [7]	29.5	29.1
PGF-SLR	27.7	28.3

**Table 3:** Comparison of component effectiveness across the three datasets.

	DI	FGL	PRE	PHOENIX14		PHOENIX14-T		CSL-Daily	
				dev (%)	test (%)	dev (%)	test (%)	dev (%)	test (%)
	baseline			20.9	21.2	20.4	20.6	29.5	29.1
**a_1**	✓			20.7	21.1	20.1	20.4	29.3	29.0
**a_2**		✓		18.1	19.1	17.7	18.0	28.6	29.0
**a_3**			✓	20.9	21.0	20.5	20.6	29.3	29.1
**a_4**	✓	✓		17.8	18.7	17.5	18.0	28.3	28.8
**a_5**	✓		✓	20.7	20.9	20.4	21.2	29.2	28.9
**a_6**		✓	✓	17.7	18.4	17.5	18.0	28.1	28.6
**PGF-SLR**	✓	✓	✓	17.5	18.2	17.3	17.7	27.7	28.3

**Table 4:** Online inference on the PHOENIX14 dataset.

Methods	Throughput	GFLOPs	WER (%)
Baseline	15.84	175.0	20.8
PGF-SLR	25.72	11.5	18.5

## Data Availability

The data presented in this study are available on request from the corresponding author, due to future publication purposes.
